# Relation between dietary cadmium intake and biomarkers of cadmium exposure in premenopausal women accounting for body iron stores

**DOI:** 10.1186/1476-069X-10-105

**Published:** 2011-12-16

**Authors:** Bettina Julin, Marie Vahter, Billy Amzal, Alicja Wolk, Marika Berglund, Agneta Åkesson

**Affiliations:** 1 Unit of Nutritional Epidemiology, Institute of Environmental Medicine, Karolinska Institutet, Stockholm, Sweden; 2 Unit of Metals and Health, Institute of Environmental Medicine, Karolinska Institutet, Stockholm, Sweden; 3 Unit of Quantitative Assessment and Evidence-Building, LA-SER Europe Ltd., London, UK

**Keywords:** Cadmium, diet, biomarkers, gastrointestinal absorption, one-compartment model

## Abstract

**Background:**

Cadmium is a widespread environmental pollutant with adverse effects on kidneys and bone, but with insufficiently elucidated public health consequences such as risk of end-stage renal diseases, fractures and cancer. Urinary cadmium is considered a valid biomarker of lifetime kidney accumulation from overall cadmium exposure and thus used in the assessment of cadmium-induced health effects. We aimed to assess the relationship between dietary cadmium intake assessed by analyses of duplicate food portions and cadmium concentrations in urine and blood, taking the toxicokinetics of cadmium into consideration.

**Methods:**

In a sample of 57 non-smoking Swedish women aged 20-50 years, we assessed Pearson's correlation coefficients between: 1) Dietary intake of cadmium assessed by analyses of cadmium in duplicate food portions collected during four consecutive days and cadmium concentrations in urine, 2) Partial correlations between the duplicate food portions and urinary and blood cadmium concentrations, respectively, and 3) Model-predicted urinary cadmium concentration predicted from the dietary intake using a one-compartment toxicokinetic model (with individual data on age, weight and gastrointestinal cadmium absorption) and urinary cadmium concentration.

**Results:**

The mean concentration of cadmium in urine was 0.18 (+/- s.d.0.12) μg/g creatinine and the model-predicted urinary cadmium concentration was 0.19 (+/- s.d.0.15) μg/g creatinine. The partial Pearson correlations between analyzed dietary cadmium intake and urinary cadmium or blood concentrations were r = 0.43 and 0.42, respectively. The correlation between diet and urinary cadmium increased to r = 0.54 when using a one-compartment model with individual gastrointestinal cadmium absorption coefficients based on the women's iron status.

**Conclusions:**

Our results indicate that measured dietary cadmium intake can reasonably well predict biomarkers of both long-term kidney accumulation (urine) and short-term exposure (blood). The predictions are improved when taking data on the iron status into account.

## Background

Although cadmium is well-known to adversely affect kidneys and bone [1-3] and proposed to possess endocrine disrupting activity [4-7], the public health consequences of exposure to cadmium are insufficiently elucidated. Cadmium accumulates in the kidney with age and urinary cadmium is considered a valid biomarker of lifetime kidney accumulation [8] and consequently used in the assessment of cadmium-induced health effects [9,10]. Since the diet is the major source (~ 99%) of cadmium exposure in the general non-smoking population [2,11], estimations of the dietary exposure rather than measurement of urinary cadmium, could open up the possibility to perform large scale epidemiological studies. Despite cadmium's dietary origin, no studies have been undertaken to validate the estimated dietary exposure in relation to biomarkers i.e. cadmium in urine or blood.

Several factors may affect how well the estimated dietary cadmium intake relates to the concentration of cadmium in urine. The time-frame of exposure assessment and the reliability of intake measurements are examples of methodological factors. Dietary and physiological factors include the bioavailability of cadmium in the diet, the rate of gastrointestinal absorption, and cadmium's half-life in the kidney [11]. For example, the gastrointestinal absorption of cadmium is generally only a few percent, but may vary considerably between individuals. The absorption is influenced by body iron status, since cadmium has high affinity for the main intestinal iron transporter [12,13], and there is a close inverse association between serum ferritin (marker of iron stores) and blood cadmium [14-17]. By accounting for factors such as gastrointestinal absorption, age, weight, and half-life, a one-compartment toxicokinetic model may serve as the link between dietary cadmium exposure and urinary cadmium excretion, and may thus provide a more reliable way of validation [11].

The aim of the present study was to assess the relationship between dietary cadmium intake and cadmium in urine and in blood in premenopausal women, exploring the influence of the physiological factors in detail. For this purpose we took advantage of the thorough information available in a previous study [15], in which cadmium intake was assessed based on duplicate diets and the individual gastrointestinal absorption could be estimated via comprehensive data on iron status.

## Methods

### Study population

Fifty seven, 20-50 year old, non-obese women who had been non-smokers for at least five years were recruited from Western Sweden as described in detail in Berglund *et al*. [15]. Informed consent was obtained from the participants and the study was approved by the Ethics Committee of Karolinska Institutet, Stockholm, Sweden.

### Assessment of cadmium in diet, urine and blood

Duplicate portions of all foods consumed during four consecutive days, were collected for assessment of the current dietary intake of cadmium and weighted and estimated dietary records were completed in parallel. Comparisons between the current consumption of different foods from the duplicate portions/diet records and that of the usual consumption as reported in a food frequency questionnaire indicated that the current intake reflected the usual dietary habits of the main food groups well. Further, no significant seasonal variations in the duplicate diets were present [15]. Cadmium in the duplicate portions was measured using flame atomic absorption spectrophotometry (detection limit 0.001-0.002 μg cadmium/g) [15].

First voided morning urine as well as a 24-hour urine sample was used for the assessment of urinary cadmium. All the materials used for sample collection, preparation and storage were acid washed with 10% HNO_3 _and tested for possible cadmium contamination. Cadmium in urine was determined by Graphite Furnace Atomic Absorption Spectrophotometry (GFAAS, method of standard addition) [15]. To correct the cadmium concentrations in the spot urine samples for the variation in dilution, each sample was adjusted for the creatinine content in urine [18]. For quality control purposes, commercial reference materials were used with satisfactory results [15]. Blood samples were collected and cadmium in blood, mainly reflecting the recent exposure [2], was measured by GFAAS with background correction [15].

### Estimation of cadmium absorption

**Figure 1:**
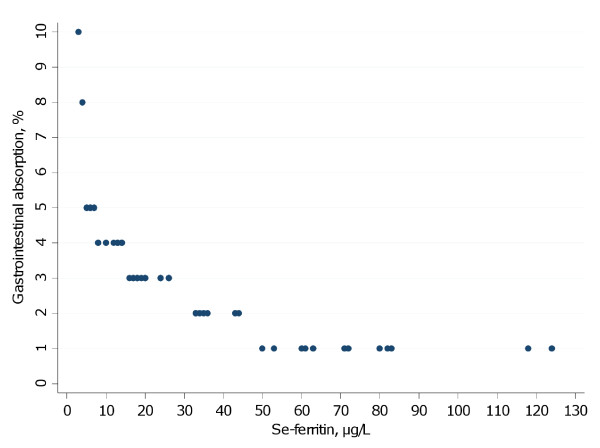
**Estimated gastrointestinal absorption of cadmium (%) based on serum ferritin levels (μg/L)**.

Blood serum was analyzed for ferritin. Low and depleted iron stores, i.e. low serum ferritin levels [19], leads to an increased absorption of cadmium from the gastrointestinal tract [14] and can be observed as an inverse relationship between cadmium concentrations in blood and serum ferritin levels [15]. To be able to use individual data on serum ferritin levels, each individual was assigned a value on the absorption coefficient (1-10%), to roughly match the observed inverse relationship between measured cadmium in blood and serum ferritin levels [15]. Participants with the lowest serum ferritin were given the highest absorption coefficient and vice versa (Figure 1).

### Toxicokinetic model

In order to quantify the relationship between cadmium intake and urinary cadmium, a one-compartment toxicokinetic model of cadmium was used, thoroughly described in Amzal *et al*. 2009 [11]. In brief: For a given intake of cadmium (*d_0_*) at time 0, the accumulated amount of cadmium in the kidney at time *t *is calculated as:

$$Cadmium_{kidney}\left(t\right)=f_{k}\times \underset{i}{\sum }\frac{d_{i}t_{1∕2}}{\log\left(2\right)}\exp\left(-\frac{\log\left(2\right)\cdot \left(t-i\right)}{t_{1∕2}}\right)$$

where *t_1/2 _*is the cadmium half-life, *d *is the dietary cadmium intake and *f_k _*is:

$$f_{k}=\frac{Absorption\times fraction_{kidney}\times coefficient_{cortex}}{Weight_{kidney}}$$

We based *f_k _*on the following biological data: 1) The gastrointestinal absorption coefficient among women was assumed to range from 1% to 10% [14,20], 2) The fraction of cadmium transported to kidney (*fraction_kidney_*) was 1/3 [21,22], 3) The coefficient (*coefficent_cortex_*) translating cadmium concentration in the whole kidney into that of the cortex was 1.25 [21-23], and 4) The kidney weight was 0.43% of total body weight [21,22]. The urinary cadmium concentration is assumed to be proportional to the cadmium concentration in the kidney cortex; 1.7 μg/g creatinine in urine reflecting 50 mg/kg in the kidney [8]. An average cadmium half-life of 11.6 years [11] and individual data for dietary cadmium exposure, age, weight and gastrointestinal absorption were used.

### Statistical methods

Pearson's correlation coefficient was used to assess univariate associations as the residuals showed no major deviation from a linear pattern. First, the mean daily dietary cadmium intake measured by cadmium in the duplicate portions was correlated with the measured urinary cadmium excretion adjusted for creatinine. In a second step, we used Pearson's partial correlation coefficients to see if age, weight and serum ferritin levels had any effects on the correlation between dietary cadmium and urinary cadmium concentrations as well as cadmium concentrations in blood. Finally, the one-compartment model was applied to compare the measured urinary cadmium with the predicted urinary cadmium from the dietary intake, assuming the same dietary intake over lifetime until the current age. All statistics were made using SAS, version 9.2 (SAS Institute Inc, Cary, NC) and graphs were made using Stata, version 11 (StataCorp, College Station TX, USA).

## Results

**Figure 2:**
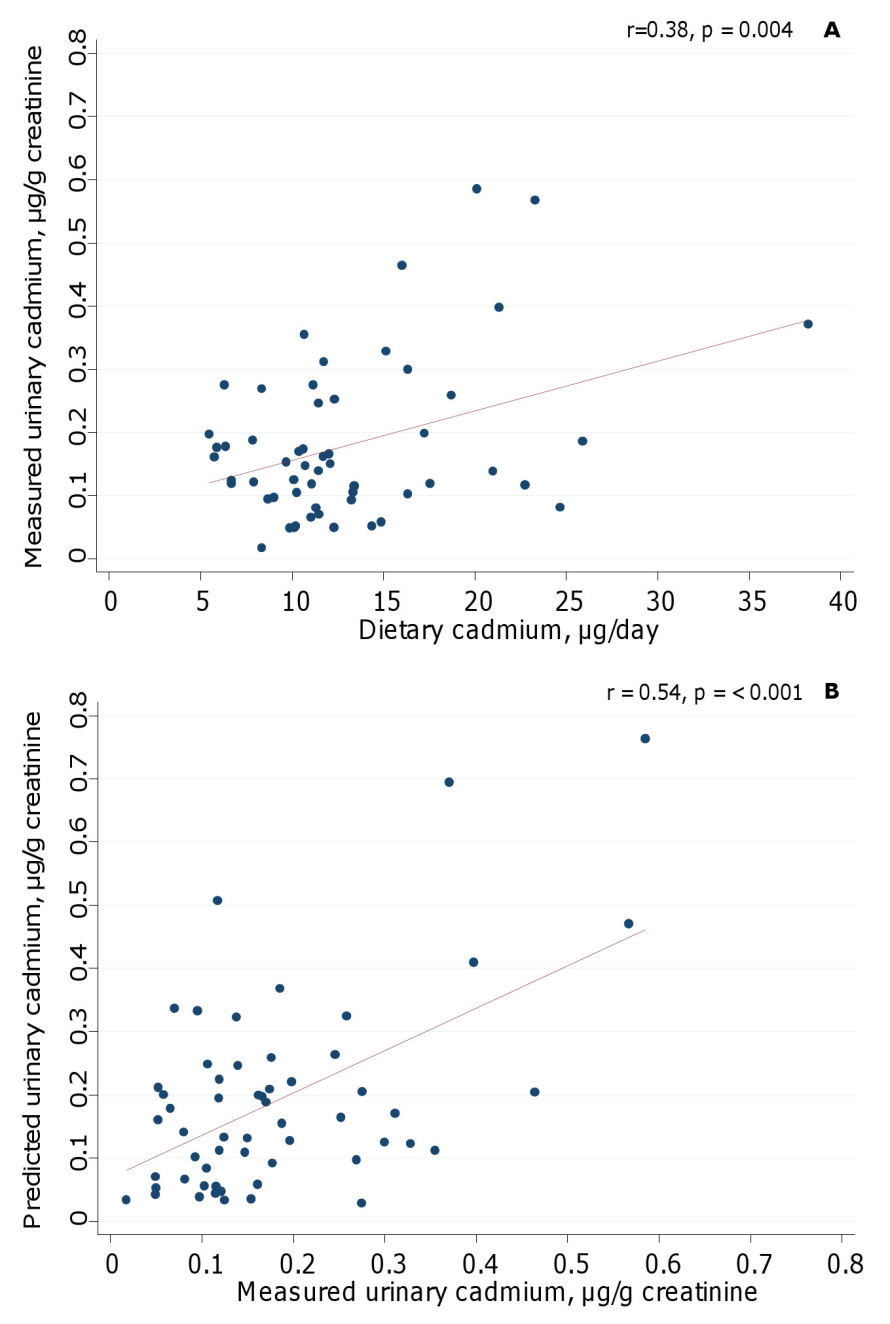
**Correlation between analyzed urinary cadmium adjusted to creatinine content in urine and: A) dietary exposure from duplicate diets and B) model-predicted urinary cadmium from duplicate diets taking individual variation in physiological factors (age, weight and gastrointestinal absorption of cadmium) into account**.

Characteristics of the participants are shown in Table 1. The Pearson correlation between dietary cadmium concentrations and urinary cadmium concentrations was 0.38 (*P *= 0.004) and is shown in Figure 2A. The results remained essentially the same (r = 0.32; *P *= 0.01) after exclusion of one woman with high cadmium intake (> 35 μg/day). As this was not considered an unreasonably high intake, all women were kept in the analyses. Similar results were obtained by replacing urinary cadmium (μg/g creatinine) in spot urine samples by the urine excreted over 24-hours (r = 0.35; *P *= 0.007) or by excluding four samples with too low urinary creatinine concentration (< 0.3 g/L; no samples were > 3 g/L), indicating that our results were fairly robust. Age, weight and serum ferritin levels had some impact on the correlation between dietary cadmium and urinary or blood cadmium concentrations; partial Pearson r = 0.43 (*P *= 0.001) for urinary cadmium and partial Pearson r = 0.42 (*P *= 0.001) for blood cadmium concentrations. The same results were obtained if weight was replaced by body mass index.

**Table 1:** Characteristics of the 57 participating women

Background variable	Mean	Median	Range
Age (y)	37	38	20-50
Body weight (kg)	62	61	47-82
Body Mass Index (kg/m^2^)	22	22	19-30
Energy intake (kcal/day)	1843	1790	1080-3276
Cadmium intake (μg/day)*	13	11	5-38
Urinary creatinine (g/L urine)	0.70	0.65	0.14-1.64
Urinary cadmium excretion (μg/g creatinine)	0.18	0.15	0.02-0.59
Blood cadmium (μg/L)	0.27	0.23	0.09-0.96
Model-predicted urinary cadmium excretion (μg/g creatinine)	0.19	0.16	0.03-0.76
Serum ferritin (μg/L)	29	18	3-124

* based on duplicate portions during 4 consecutive days

The measured concentration of cadmium in urine (mean 0.18 ± 0.12 μg/g creatinine) was very similar to that predicted by the one-compartment model based on the measured dietary cadmium intake (mean 0.19 ± 0.15 μg/g creatinine). The Pearson correlation between the measured and predicted urinary cadmium was r = 0.54 (*P *= < 0.001; Figure 2B). Thus, by assigning individual absorption coefficients based on the women's iron status, the association between dietary exposure and urinary cadmium concentration was improved.

## Discussion

In this study of premenopausal women we found that dietary cadmium intake was clearly related to biomarkers of both long-term and recent exposure. The strongest correlation was observed between dietary cadmium exposure and urinary cadmium when individual gastrointestinal absorption of cadmium was considered in a one-compartment toxicokinetic model.

In the present study, the mean concentration of the measured urinary cadmium (0.18 μg/g creatinine) was very similar to the model-predicted concentration of urinary cadmium (0.19 μg/g creatinine), supporting the accuracy of the model. We have previously demonstrated that the one-compartment toxicokinetic model performed well in predicting the mean urinary cadmium from dietary assessed cadmium in a population of postmenopausal Swedish women [11]. We based the study on premenopausal women, known to have greater variability in toxicokinetics of cadmium due to a higher prevalence of low iron stores [24] and a generally higher cadmium body burden than men. Whether similar associations are present among men remain to be evaluated. The correlation between cadmium in duplicate portions and urinary cadmium concentration observed in the present study is in agreement with that observed in women from Japan (r = 0.39) [25]. However, in the present study we also took into account the individual gastrointestinal absorption and the results clearly illustrates how this increases the correlation (r = 0.38 vs. r = 0.54).

There may, however, be several factors contributing to a distortion of the association between dietary cadmium intake and the concentration in urine. Data on time trends in food give little support for any changes in cadmium concentrations [26]. Under the assumption that the food habits of the study participants have not changed dramatically, which is supported by the questionnaire completed prior to the study, we consider the dietary cadmium exposure to be fairly stable over time. Thus, the non-smoking women in the present study can be assumed to be close to steady state in regard to their cadmium intake and renal accumulation of the metal, as they probably have been exposed to similar dietary cadmium levels during their entire life. Although urinary cadmium is considered a good biomarker of the long-term kidney accumulation, it may be influenced by several factors related to either the kidney cadmium accumulation such as the proportion of cadmium transferred to the kidney [21], the subsequent half-life in the kidney [20] or to the method used to compensate for the variation in urine dilution such as creatinine excretion or the urinary density [18,27]. All these factors will eventually lead to variability in the urinary cadmium concentration that is unrelated to the intake. Furthermore, the urinary cadmium concentrations in this study were low and it is not clear whether the high correlation between urinary cadmium concentration and the kidney cadmium accumulation (i.e. 1.7 μg/g creatinine reflects 50 mg/kg in the kidney) observed at autopsy at much higher concentrations [8] is equally valid. The net effect of these sources of variation is to weaken correlations between the dietary intake assessment and the biomarker. For this reason it is likely that the magnitude of such correlations will tend to be rather modest, even when the dietary measurements, analyzed and/or reported, are highly accurate and precise [28]. Collection of duplicate portions is known to influence dietary habits [29]. Although there was a good agreement between the duplicate portions and the participants' general dietary habits as reflected by the food frequency questionnaire [15], a lower total dietary intake of approximately 10-20% in these women was indicated according to a previous validation against biomarkers of protein intake [30]. However, in the present study, the partial correlation between dietary cadmium intake and urinary concentrations did not differ from the correlation between dietary cadmium and blood cadmium concentrations. Thus, the current dietary cadmium intake seemed to well represent the long-term intake.

Several factors may affect cadmium absorption and accumulation in the body. In the present study we were able to adjust for age, iron status and body weight. Also parity may affect the cadmium absorption as it has been shown that urinary cadmium concentrations are increased with each full-term pregnancy [17]. Thus, adjusting the model for parity could have improved the correlation between dietary cadmium intake and the biomarkers. Unfortunately, we did not have such information. We analyzed the total dietary cadmium intake in the present study. As the absorption of cadmium may vary between different foods depending on cadmium's chemical form and binding, further adjustment by specific foods may have affected the correlation. Tofu, for example, has been shown to increase the urinary cadmium concentration [31], but was rarely consumed in these women.

Our results are limited by the modest sample size. Also the external validity may be limited since this study was not aimed to be representative of the population. However, the prevalence of depleted iron stores [32] as well as the cadmium intake [33] in these women was very similar to that reported in other Swedish populations, indicating that the women were not different with respect to the major aspects under investigation. As the study is restricted to women we cannot make any inference to men with respect to the observed correlations. There is, however, no obvious reason to believe that the results would be different in men. In addition, the very low urinary cadmium concentrations (range 0.02-0.6 μg/g creatinine) observed in some women may result in a greater uncertainty which could underestimate the associations. Still the observed correlations were reasonably high.

## Conclusions

Our results indicate that estimations of the dietary cadmium intake can reasonably well predict biomarkers of both short-term exposure and long-term kidney accumulation. The prediction is improved by including information on the iron status.

## Abbreviations

s.d.: standard deviation; HNO_3_: nitric acid; GFAAS: Graphite Furnace Atomic Absorption Spectrophotometry; r: Pearson correlation coefficient.

## Competing interests

The authors declare that they have no competing interests.

## Authors' contributions

BJ participated in the design of the study and performed the statistical analysis, interpreted the data and drafted the manuscript. MV, MB and AÅ have made substantial contributions to the original conception and design of the duplicate portion study, acquisition of data and contributed to the manuscript. AÅ and AW conceived of the study and participated in its design and helped to draft the manuscript. BA contributed to the one-compartment model and contributed to the manuscript. All authors have read and approved the final manuscript.
